# Prediction of early mucosal healing of Crohn’s disease after treatment with biologics- a novel nomogram based on radiomics and clinical risk factors

**DOI:** 10.3389/fphar.2025.1586300

**Published:** 2025-05-23

**Authors:** Linlin Huang, Hui Li, Shuo Wang, Ying Ren

**Affiliations:** ^1^ Department of Radiology, Shengjing Hospital of China Medical University, Shenyang, China; ^2^ Department of Digestive, Shengjing Hospital of China Medical University, Shenyang, China

**Keywords:** Crohn’s disease, mucosal healing, biologics, computed tomography enterography, radiomics

## Abstract

**Background:**

Predicting endoscopic remission is crucial for optimizing clinical treatment strategies and switching biologics in Crohn’s disease (CD). Mucosal healing (MH) is a key therapeutic target. This study aimed to develop a clinically applicable prediction model for early MH in CD patients receiving biological therapy.

**Methods:**

This study retrospectively analyzed 120 CD patients diagnosed between 2018 and 2023, randomly divided into a training cohort and an internal validation cohort 1. Additionally, 34 prospectively enrolled CD patients diagnosed between 2024 and 2025 formed an internal validation cohort 2. Clinical indicators and conventional imaging features were evaluated to establish a clinical model. Radiomics features were extracted from computed tomography enterography (CTE) images, with regions of interest (ROIs) manually delineated to align with ulcerated intestinal segments identified through colonoscopy. A radiomics model was constructed, and a radiomics score (Rad-score) was derived. A clinical-radiomics nomogram was then developed by integrating Rad-score with clinical risk factors. Model performance was assessed using discrimination, calibration, decision curve analysis (DCA), and clinical impact curves.

**Results:**

The clinical-radiomics nomogram demonstrated strong predictive performance, with AUC values of 0.948 (95% CI: 0.902–0.995) in the training cohort, 0.925 (95% CI: 0.805–1.0) in the internal validation cohort 1, and 0.940 (95% CI: 0.802–0.993) in the internal validation cohort 2. The nomogram outperformed standalone clinical and radiomics models, with DCA confirming its clinical utility.

**Conclusion:**

The developed nomogram effectively predicts early MH in CD patients undergoing biological therapy, providing a practical tool for clinicians to optimize treatment strategies and improve outcomes.

## 1 Introduction

Crohn’s disease, a chronic inflammatory condition affecting the gastrointestinal tract, poses substantial physical and mental burdens on patients due to its persistent nature and potential complications (Torres et al., 2017; Roda et al., 2020). In recent years, biological agents such as Infliximab (IFX) and Ustekinumab (UST), has been proved to be more effective in promoting MH (Leal et al., 2015; McDonald et al., 2023), which has become a very important treatment objective (Pineton de Chambrun et al., 2010; Sakurai et al., 2017). However, there are still many patients who suffer treatment failure with biological agents (Dalal et al., 2021; Zhang et al., 2021), and surgical intervention is still unavoidable finally after high cost on hospitalization and treatment (Chirra et al., 2023). Early prediction of MH to biological therapy before initiation of treatment is in great need to aid clinicians in deciding personalized treatment strategies.

Endoscopy remains the gold standard for evaluating MH (Núñez et al., 2021). Endoscopy is not only invasive and operator-dependent, limited to visualizing the intestinal lining, but the information it provides is also limited and subjective in predicting treatment efficacy. Radiological examination, particularly CTE, is more preferred due to its non-invasiveness, rapid scanning speed, and widespread applicability. CTE not only correlates well with endoscopic results but also effectively shows the imaging features of CD beyond endoscopy, including the thickness and enhancement pattern of intestinal wall, peri-intestinal lymph nodes (Sakurai et al., 2017; Noh et al., 2020; Rong et al., 2023), and extraintestinal complications such as fistula and abscess (Bruining et al., 2018; Minordi et al., 2022). In traditional CTE examinations, evaluating intestinal diseases largely relies on the subjective interpretation of radiologists, increasing the possibility of explanatory variation (Alyami, 2023). Radiomics is the process of converting digital medical images into high-dimensional data that can be mined (Gillies et al., 2016). It includes extracting features from medical images to construct predictive models, which may help to predict results of interest. And we can also combine radiomics data with other patient clinical information to enhance the predictive ability of decision support models (Gillies et al., 2016; Lambin et al., 2017). In the field of oncology, radiomics has made significant progress (Lambin et al., 2012). In recent years, radiomics has been investigated in the field of CD, allowing radiologists to evaluate intestinal characteristics more objectively and reduce variability between observers. In this study, we tried to establish a clinical-radiomics nomogram utilizing radiomics from CTE combined with clinical features to predict early endoscopic MH after biologic therapy for Crohn’s disease.

## 2 Materials and methods

### 2.1 Patients and study design

**FIGURE 1:**
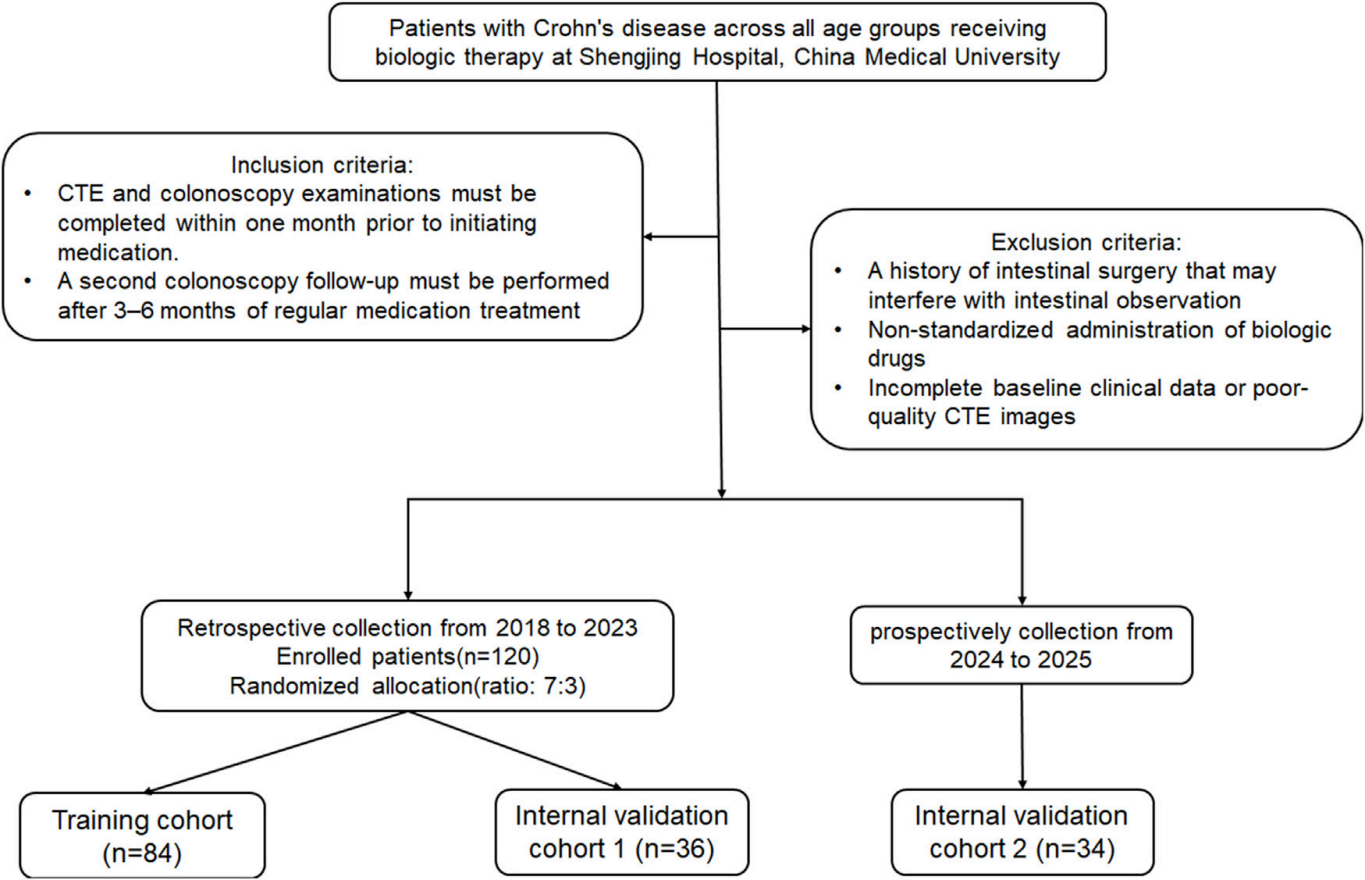
Flowchart of patient recruitment pathway.

This retrospective study was approved by the Institutional Review Committee (Protocol No. 2023PS1412K). The study included patients of all age groups with CD who underwent biologic therapy at our hospital from 2018 to 2025, and who had no other treatment modalities besides biologic agents. The diagnosis of CD adhered to the ECCO-ESGAR Guideline (Maaser et al., 2019). Patients received regular injections of two biological agents, Infliximab or Ustekinumab. The inclusion criteria were: (1) CTE and colonoscopy performed within 1 month prior to drug treatment, and (2) perform a second colonoscopy review after 3–6 months of regular medication use. Exclusion criteria included: (1) Previous history of intestinal surgery affecting intestinal observation; (2) Irregular drug injections; (3) Incomplete clinical baseline data or poor CT image quality. Early MH was defined as the complete resolution of mucosal ulcers observed during baseline colonoscopy after short-term (3–6 months) of medication therapy (Peyrin-Biroulet et al., 2014; Bertani et al., 2020). The patients were randomly divided into a training cohort and an internal validation cohort 1 in a 7:3 ratio, and 34 CD patients from 2024 to 2025 were prospectively collected as an internal validation cohort 2. Baseline clinical data included age, gender, serum albumin, erythrocyte sedimentation rate, C-reactive protein (CRP) levels, disease behavior, and disease location classified by Montreal. Imaging features, such as intestinal wall thickness, mural stratification, intestinal narrowing, comb sign, lymph node diameter, and perianal lesions, were obtained from each patient’s CT images. The recruitment process for these patients is illustrated in Figure 1.

### 2.2 CT examination and image evaluation

All patients underwent standardized preparation before CTE examination. Each patient needs to fast for 12 h before CTE examination and take 1500 mL of polyethylene glycol solution orally the night before to maintain gastrointestinal patency and cleanliness. Each patient underwent a plain and enhanced CT scan of the entire abdomen (the scanning equipment was either Philips Brilliance 128 or Siemens Definition 64). The scanning conditions were as follows: tube current of 250 mA, tube voltage of 120 kV, slice thickness of 3 mm, slice interval of 3 mm, field of view of 500 mm × 500 mm, and matrix of 512 × 512. The contrast agent used for intravenous injection was an iodinated contrast agent with an iodine concentration of 320 mg/mL. The injection dose was 1.5 mL/kg, and the injection rate was 2.0–2.5 mL/s. The arterial and venous phases were scanned at 25–30 s and 60–70 s after the injection of the contrast agent, respectively. Two radiologists, each with over 5 years of experience in abdominal imaging diagnosis, independently evaluated the radiological findings. The assessment included intestinal wall thickness (maximum thickness at the lesion site≥10 mm), short-axis diameter of adjacent mesenteric lymph nodes (≥10 mm), the 10 mm cut-off value was selected in accordance with well-established criteria from previous authoritative studies (Song et al., 2024),intestinal wall stratification (present or absent), intestinal stenosis (confirmed by endoscopy), comb sign (present or absent), and perianal lesions (present or absent). Quantitative indicators were calculated as the average of the two radiologists' measurements, while qualitative indicators were resolved through discussion to reach a consensus in cases of disagreement. Conventional imaging features with consensus were used as clinical risk factors for further analysis.

### 2.3 Three-dimensional segmentation and radiomics feature extraction

Arterial and venous phase images of CTE were used for delineating the regions of interest (ROIs) and extracting lesion features. Two radiologists, each having over 5 years of experience, drew the ROIs. The three-dimensional (3D) segmentation of each lesion was carried out utilizing 3D-slicer software. The criteria for selecting the ROIs were defined as follows: the inflammatory intestinal segment corresponding to the evident ulcer identified during the baseline endoscopic examination. To avoid volume effects, the ROIs should be delineated as far away from the outer edge of the intestinal wall as possible, avoiding the inclusion of gas, feces, and blood vessels within the lumen. Subsequently, Python software (v.2.7.0) (available at https://www.python.org) was employed to extract intestinal wall features segmented under Gaussian filters, including shape features, texture features, and wavelet transform. The inter observer reliability and intra observer repeatability of radiomics features are evaluated using intraclass correlation coefficients (ICCs). The method involves two radiologists randomly selecting 50 patient images for ROIs delineation 2 weeks after the initial delineation, and calculating the intra group correlation coefficient.

### 2.4 Construction of the clinical model

Univariate analysis was conducted to compare the clinical characteristics of patients in the MH group and the non-MH group. Following that, statistically significant clinical information and routine imaging features were subjected to multiple logistic regression analysis (P < 0.05), and the odds ratio (OR) of each independent factor was calculated as the relative risk estimate for the 95% confidence interval (CI). Finally, the identified clinical risk factors will be used to establish a clinical prediction model through logistic regression.

### 2.5 Construction of the radiomics model

**FIGURE 2:**
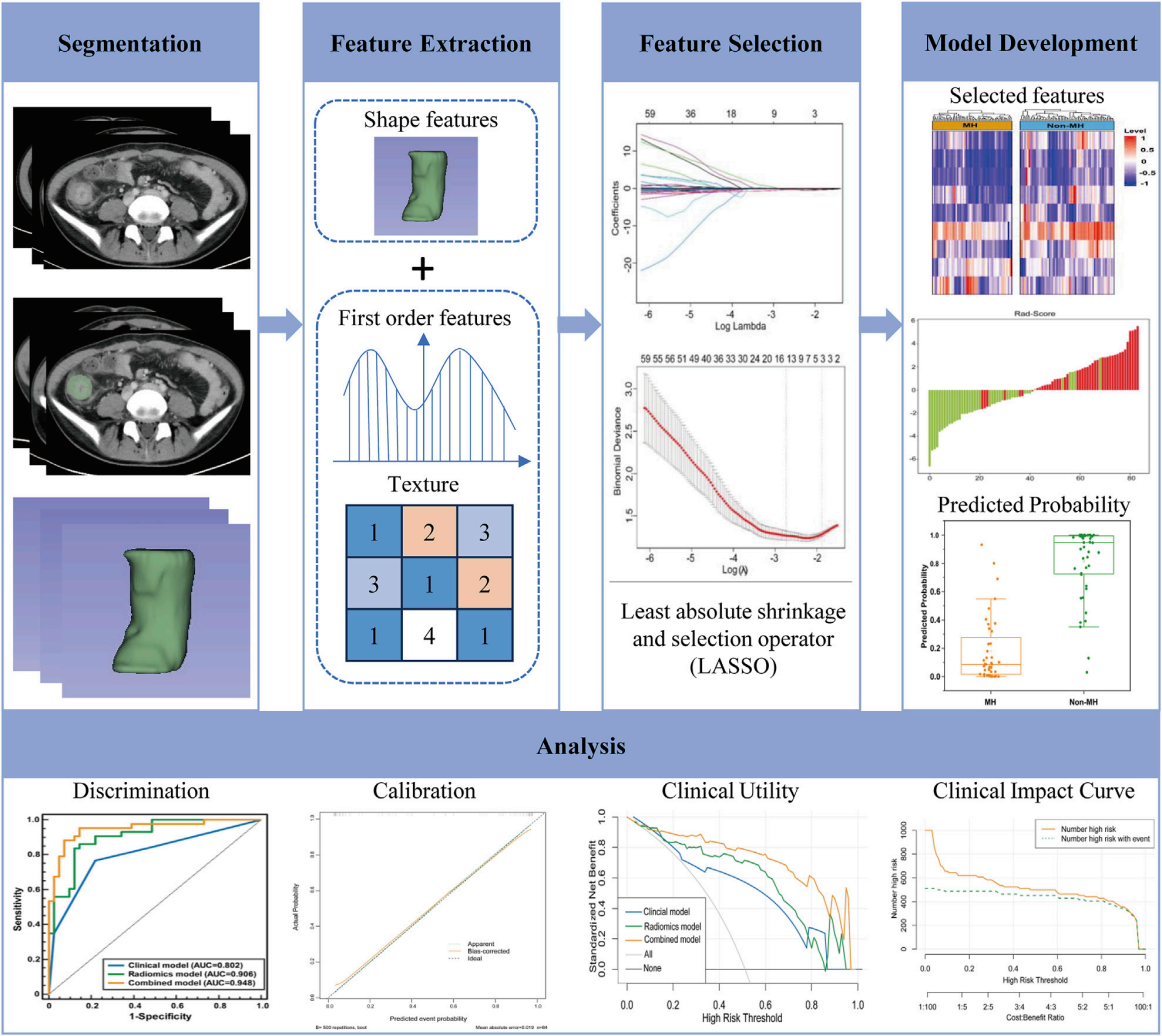
The radiomics flow chart of the study.

First, Z-score normalization was applied to standardize the 1,327 radiomics features extracted from CTE, enhancing data comparability. The formula used was **Z = (x-μ)/σ**, where *μ* represents the mean of the entire dataset and *σ* denotes the standard deviation. Next, an independent sample t-test was performed to identify features associated with treatment response (P < 0.05). Subsequently, Spearman correlation analysis was conducted to exclude features with correlation coefficients exceeding 0.95. Following this, the Least Absolute Shrinkage and Selection Operator (LASSO) regression with 10-fold cross-validation was employed to further eliminate redundant features. This process retained 9 features, including 5 from the arterial phase (AP) and 4 from the venous phase (VP). A radiomics score (Rad-Score) was then calculated using logistic regression based on the selected features. The workflow of the radiomics prediction model and research flowchart is illustrated in Figure 2.

### 2.6 Construction of the clinical radiomics nomogram

The clinical radiomics nomogram was constructed by integrating clinical features obtained from multiple logistic regression (p < 0.05) with the Rad-score. Calibration curves were utilized to assess the calibration nomogram. The clinical model, radiomics model, and clinical radiomics nomogram were evaluated based on the receiver operating characteristic (ROC) curve and the area under the curve (AUC). Decision Curve Analysis (DCA) quantified the net benefits of training and testing by combining models under different threshold probabilities. Clinical Impact Curve (CIC) is used to evaluate the accuracy of three models.

### 2.7 Statistical analysis

Statistical analyses were conducted using R statistical software (version 3.6.3; www.R-project.org), SPSS (version 25.0, IBM, Armonk, NY, United States), and MedCalc (version 19.6.1, MedCalc Software BVBA, Ostend, Belgium). For normally distributed quantitative data, independent sample t-tests were used to compare differences between groups. For non-normally distributed quantitative data, the Mann-Whitney U test was applied, while categorical data were compared using the chi-square test or Fisher’s exact test, as appropriate. Differences in AUC values between models were assessed using DeLong test. A two-tailed P-value <0.05 was considered statistically significant.

## 3 Results

### 3.1 Construction and efficiency of the clinical prediction model

**FIGURE 3:**
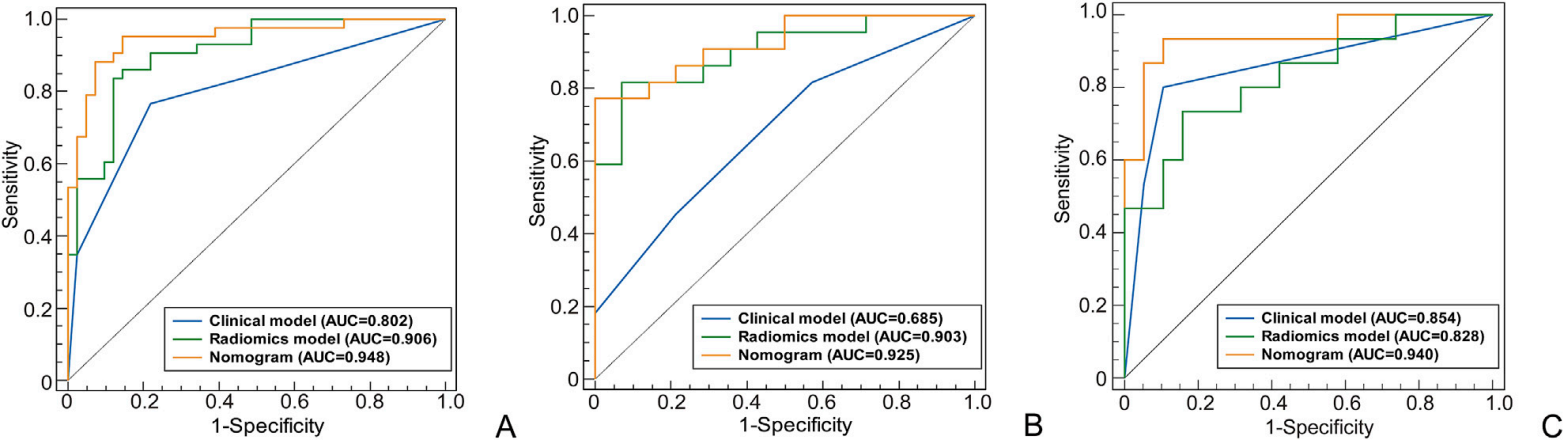
The ROC curves of different models used to identify the MH or Non-MH. [**(A)** Training cohort; **(B)** Internal validation cohort 1; **(C)** Internal validation cohort 2].

A total of 120 patients were included, with 55 achieving early MH after drug treatment, while 65 did n’t. Among them, there are 95 male patients and 25 female patients. The detailed information regarding clinical and imaging data for all patients is presented in Table 1. Multiple logistic regression identified intestinal wall thickness and lymph node diameter in imaging as clinical risk factors (p < 0.05, Table 2). Other clinical data include age, gender, serum albumin, erythrocyte sedimentation rate, C-reactive protein (CRP) levels, disease behavior, and disease location classified by Montreal and imaging features, such as mural stratification, intestinal narrowing, comb sign and perianal lesions (p > 0.05). This implies intestinal wall thickness ≥10 mm and lymph node diameter ≥10 mm are factors suggesting difficulty in achieving early endoscopic MH after biological therapy. A clinical prediction model using the above two risk factors were established. There were no significant differences in the clinical and imaging date between the training and internal validation cohorts (p > 0.05, Table 3). In the training cohort, the AUC value, sensitivity, specificity, and accuracy for predicting whether MH would be achieved after short-term biological therapy were 0.802 (95% confidence interval: 0.710–0.894), 0.780, 0.767, and 0.774, respectively. In the internal validation cohort 1, the AUC value, sensitivity, specificity, and accuracy were 0.685 (95%CI: 0.517–0.853), 0.429, 0.818, and 0.667, respectively. In the internal validation cohort 2, the AUC value, sensitivity, specificity, and accuracy were 0.854 (95%CI: 0.691–0.951), 0.800, 0.895, and 0.853, respectively (Table 4; Figure 3).

**TABLE 1:** Comparison of the clinical data and image features between the MH and Non-MH groups of Crohn’s disease patients.

Clinical factors	MH (N = 55)	Non- MH (N = 65)	Overall (N = 120)	P-value
Gender				0.487
Male	42 (76.4%)	53 (81.5%)	95 (79.1%)	
Female	13 (21.8%)	12 (18.5%)	25 (20.8%)	
Age	25.0 (19.0–39.0)	26 (20.0–33.0)	25.5 (20.0–34.0)	0.804
CRP (mg/dL)	10.0 (3.78–21.1)	28.8 (13.1–57.0)	16.1 (7.7–46.6)	<0.001
ESR (mm/h)	19.0 (8.0–40.0)	34.0 (18.5–58.5)	26.0 (13.25–48.6)	<0.001
Albumin (g/dL)	37.5 ± 5.4	35.8 ± 5.1	36.5 ± 5.2	0.083
Types of drugs				0.150
Infliximab	32 (58.2%)	46 (70.8%)	78 (65.0%)	
Ustekinumab	23 (41.9%)	19 (29.2%)	42 (35.0%)	
Montreal location classifcation, n (%)				0.004
L1 (ileal disease)	11 (20.0%)	1 (1.5%)	12 (10.0%)	
L2 (colonic disease)	4 (7.4%)	9 (13.8%)	13 (10.8%)	
L3 (ileocolonic disease)	49 (89.1%)	55 (84.6%)	104 (86.7%)	
Montreal behavior classifcation, n (%)				0.157
B1 (non stricturing, non-penetrating)	30 (54.5%)	24 (36.9%)	54 (45.0%)	
B2 (stricturing)	23 (41.8%)	38 (58.5%)	61 (50.8%)	
B3 (penetrating)	9 (3.6%)	10 (4.6%)	19 (15.8%)	
Imaging characteristics, n (%)				
Intestinal wall thickness (≥10 mm)	14 (25.5%)	45 (69.2%)	59 (49.2%)	<0.001
Mural stratifcation	26 (47.2%)	51 (78.4%)	77 (64.2%)	<0.001
Intestinal narrowing	23 (41.8%)	38 (58.5%)	61 (50.8%)	0.069
Comb sign	51 (92.7%)	61 (93.8%)	112 (93.3%)	1.00
Lymph node diameter (≥10 mm)	14 (25.5%)	28 (43.1%)	42 (35.0%)	0.044
Perianal lesions	25 (45.6%)	31 (47.7%)	56 (46.7%)	0.807

Data are expressed as median (IQR) or number (percentage).

Abbreviations: CRP, C-reactive protein; ESR, erythrocyte sedimentation rate.

**TABLE 2:** Multivariate logistic regression analysis.

Clinical factors	OR (95%CI)	P-value
CRP (mg/dL)	0.997 (0.981–1.013)	0.715
ESR (mm/h)	0.980 (0.958–1.003)	0.078
Intestinal wall thickness (≥10 mm)	4.636 (1.708–12.583)	0.003
Lymph node diameter (≥10 mm)	3.141 (1.182–8.348)	0.022
Montreal location classifcation, n (%)	0.600 (0.126–2.866)	0.522
Mural stratifcation	1.261 (0.435–3.657)	0.669

**TABLE 3:** Clinical and imaging characteristics of patients in the training and internal validation cohorts.

Clinical factors	Training (N = 84)	Testing (N = 36)	Overall (N = 120)	P-value
Gender				0.462
Male	68 (81.0%)	27 (75.0%)	95 (79.1%)	
Female	16 (19.0%)	9 (25.0%)	25 (20.8%)	
Age	27.5 (20.0–34.8)	24.5 (19.25–33.0)	25.5 (20.0–34.0)	0.610
CRP (mg/dL)	17.1 (8.0–50.3)	14.75 (7.4–36.3)	16.1 (7.7–46.6)	0.523
ESR (mm/h)	27.0 (14.25–50.5)	24.0 (9.25–40.8)	26.0 (13.25–48.6)	0.345
Albumin (g/dL)	36.6 ± 5.5	36.4 ± 4.7	36.5 ± 5.2	0.888
Types of drugs				0.867
Infliximab	55 (65.5%)	23 (63.9%)	78 (65.0%)	
Ustekinumab	29 (34.5%)	13 (50.0%)	42 (35.0%)	
Montreal location classifcation, n (%)				0.118
L1 (ileal disease)	11 (13.1%)	1 (2.8%)	12 (10.0%)	
L2 (colonic disease)	10 (11.9%)	2 (5.6%)	12 (10.0%)	
L3 (ileocolonic disease)	63 (75.0%)	33 (91.7%)	96 (80.0%)	
Montreal behavior classifcation, n (%)				0.518
B1 (non stricturing, non-penetrating)	40 (47.6%)	14 (38.9%)	54 (45.0%)	
B2 (stricturing)	39 (46.4%)	22 (61.1%)	61 (50.8%)	
B3 (penetrating)	14 (16.7%)	9 (25.0%)	23 (19.2%)	
Imaging characteristics,n (%)				
Intestinalwall thickness (≥10 mm)	42 (50.0%)	17 (47.2%)	59 (49.1%)	0.780
Mural stratifcation	51 (60.7%)	26 (72.2%)	77 (64.2%)	0.228
Intestinal narrowing	39 (46.4%)	22 (61.1%)	61 (50.8%)	0.140
Comb sign	79 (94.0%)	33 (91.7%)	112 (93.3%)	0.695
Lymph node diameter (≥10 mm)	29 (34.5%)	13 (36.1%)	42 (35.0%)	0.867
Perianal lesions	44 (52.4%)	12 (33.3%)	56 (46.7%)	0.055

**TABLE 4:** Performance of the clinical model, radiomics model, and clinical radiomics model in the training and validation tests.

Variable	Group	AUC	(95% CI)	Sensitivity	Specificity	Accuracy	P-value
Clinical model	Training	0.802	0.710–0.894	0.780	0.767	0.774	0.000
	Internal validation 1	0.685	0.517–0.853	0.429	0.818	0.667	0.005
	Internal validation 2	0.854	0.691–0.951	0.800	0.895	0.853	0.249
Radiomics model	Training	0.906	0.844–0.998	0.837	0.878	0.845	0.051
	Internal validation 1	0.903	0.805–1.000	0.818	0.929	0.778	0.438
	Internal validation 2	0.828	0.660–0.935	0.733	0.842	0.828	0.165
Clinical Radiomics Nomogram	Training	0.948	0.902–0.995	0.884	0.927	0.893	
	Internal validation 1	0.925	0.845–1.000	0.773	1.000	0.833	
	Internal validation 2	0.940	0.802–0.993	0.933	0.895	0.912	

AUC, area under the curve; 95% CI, 95% confidence interval; P-value refers to the comparison of AUC, values between Clinical model, Radiomics model, and Clinical Radiomics Nomogram. The P-values (Delong test) reflect the statistical significance of AUC, differences when comparing the Clinical model and Radiomics model individually against the Clinical Radiomics Nomogram in the training and validation cohorts.

### 3.2 Construction and efficiency of the radiomics model

**FIGURE 4:**
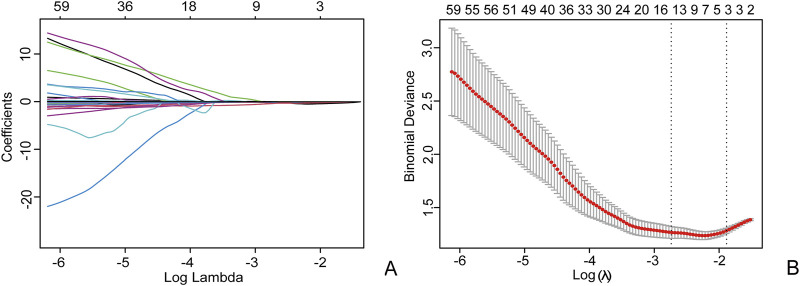
Using the Least Absolute Shrinkage and Selection Operator (LASSO) logistic regression to select radiomics features, in the LASSO model **(A)**, 10-fold cross validation is used to select the best model parameters (λ), and a dashed vertical line **(B)** is drawn at the optimal λ value, resulting in 9 radiomics features.

The intra group correlation coefficient (ICCs = 0.92) of radiomics features indicates that the extracted radiomics features have repeatability and good consistency within and between observers. Based on the LASSO algorithm, a total of 9 non-zero coefficient features were obtained (Figure 4). The radiomics model was constructed using 5 arterial phase features and 4 venous phase features after feature screening. Based on the most valuable features mentioned above, the Rad-score was calculated using the following formula:

$$\text{Radiomics model score}\left(\text{Rad}-\text{Score}\right)=-1.2429$$

$$+0.0283\times \text{original}\_\text{gldm}\_\text{DependenceVariance}\left(\text{AP}\right)$$

$$+0.0006\times \text{wavelet}.\text{HLL}\_\text{gldm}\_\text{LargeDependenceHighGrayLevelEmphasis}\left(\text{AP}\right)$$

$$+0.0265l\times \text{og}.\text{sigma}.3.0.\text{mm}.3D\_\text{glrlm}\_\text{LongRunHighGrayLevelEmphasis}\left(\text{AP}\right)$$

$$-0.1845\times \text{original}\_\text{shape}\_\text{Sphericity}\left(\text{AP}\right)$$

$$+0.1098\times \log.\text{sigma}.5.0.\text{mm}.3D\_\text{glszm}\_\text{GrayLevelNonUniformity}\left(\text{AP}\right)$$

$$-0.6368\times \log.\text{sigma}.1.0.\text{mm}.3D\_\text{glrlm}\_\text{RunEntropy}\left(\text{VP}\right)$$

$$+1.7019\times \log.\text{sigma}.1.0.\text{mm}.3D\_\text{glrlm}\_\text{RunVariance}\left(\text{VP}\right)$$

$$-0.1132\times \log.\text{sigma}.4.0.\text{mm}.3D\_\text{first}\_\text{order}90\text{Percentile}\left(\text{VP}\right)$$

$$-0.0006\times \text{wavelet}.\text{LLL}\_\text{first}\_\text{orderVariance}\left(\text{VP}\right)$$

**FIGURE 5:**
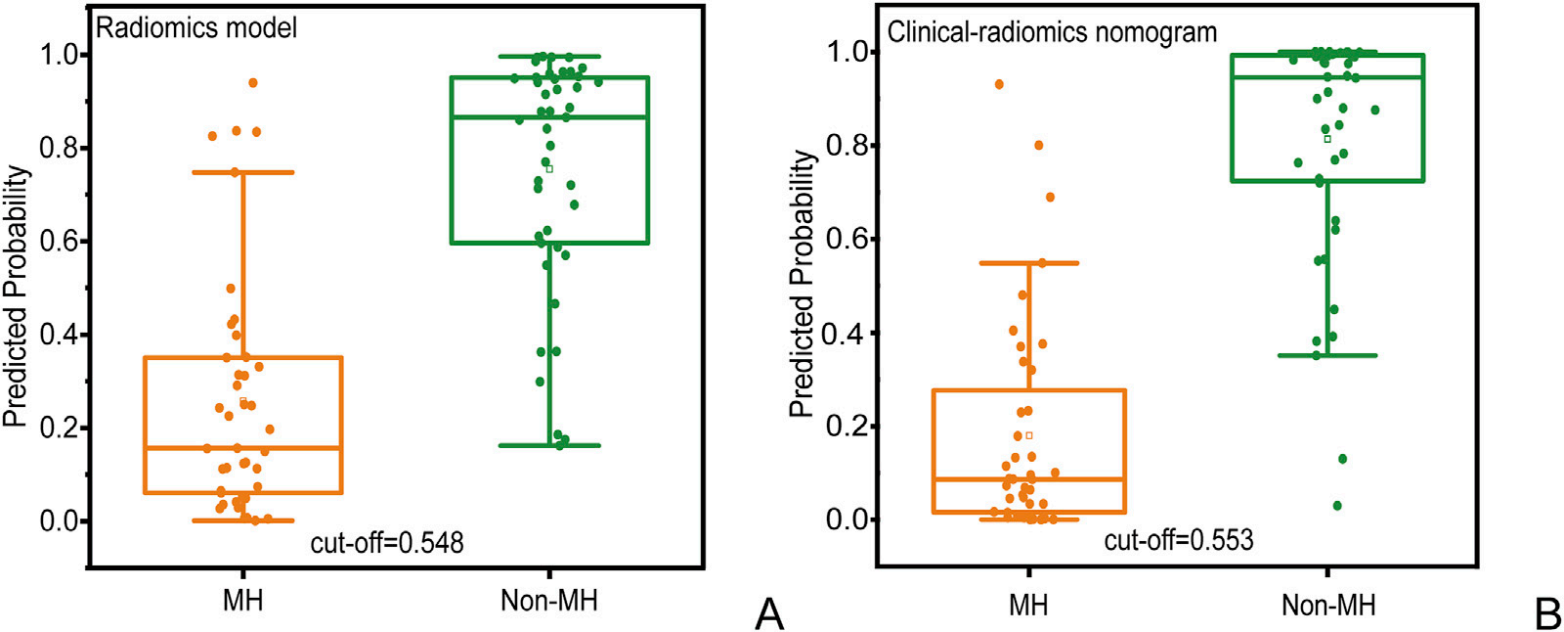
Prediction probability scatter plot of **(A)** radiomics prediction model and **(B)** clinical radiomics nomogram used to distinguish MH and Non-MH.

In the radiomics model, the critical value for distinguishing MH from Non-MH is 0.548 (Figure 5A). The AUC value, sensitivity, specificity, and accuracy for predicting early MH under endoscopy in the training cohort were 0.906 (95% confidence interval: 0.844–0.998), 0.837, 0.878, and 0.845, respectively. In the internal validation cohort 1, the AUC values, sensitivity, specificity, and accuracy were 0.903 (95% confidence interval: 0.805–1), 0.818, 0.929, and 0.778, respectively. In the internal validation cohort 2, the AUC value, sensitivity, specificity, and accuracy were 0.828 (95%CI: 0.660–0.935), 0.733, 0.842, and 0.706, respectively (Table 4; Figure 3).

### 3.3 Construction and efficiency of the clinical radiomics nomogram

A clinical radiomics nomogram based on clinical risk factors (intestinal wall thickness and lymph node diameter) and Rad-score was constructed, and the clinical radiomics nomogram score was calculated based on multivariate logistic regression analysis, as described by the formula:

Nomogram−score=2.452×Intestinal wall thickness+0.819×Lymph node diameter+5.717×Rad−Score−4.256.

**FIGURE 6:**
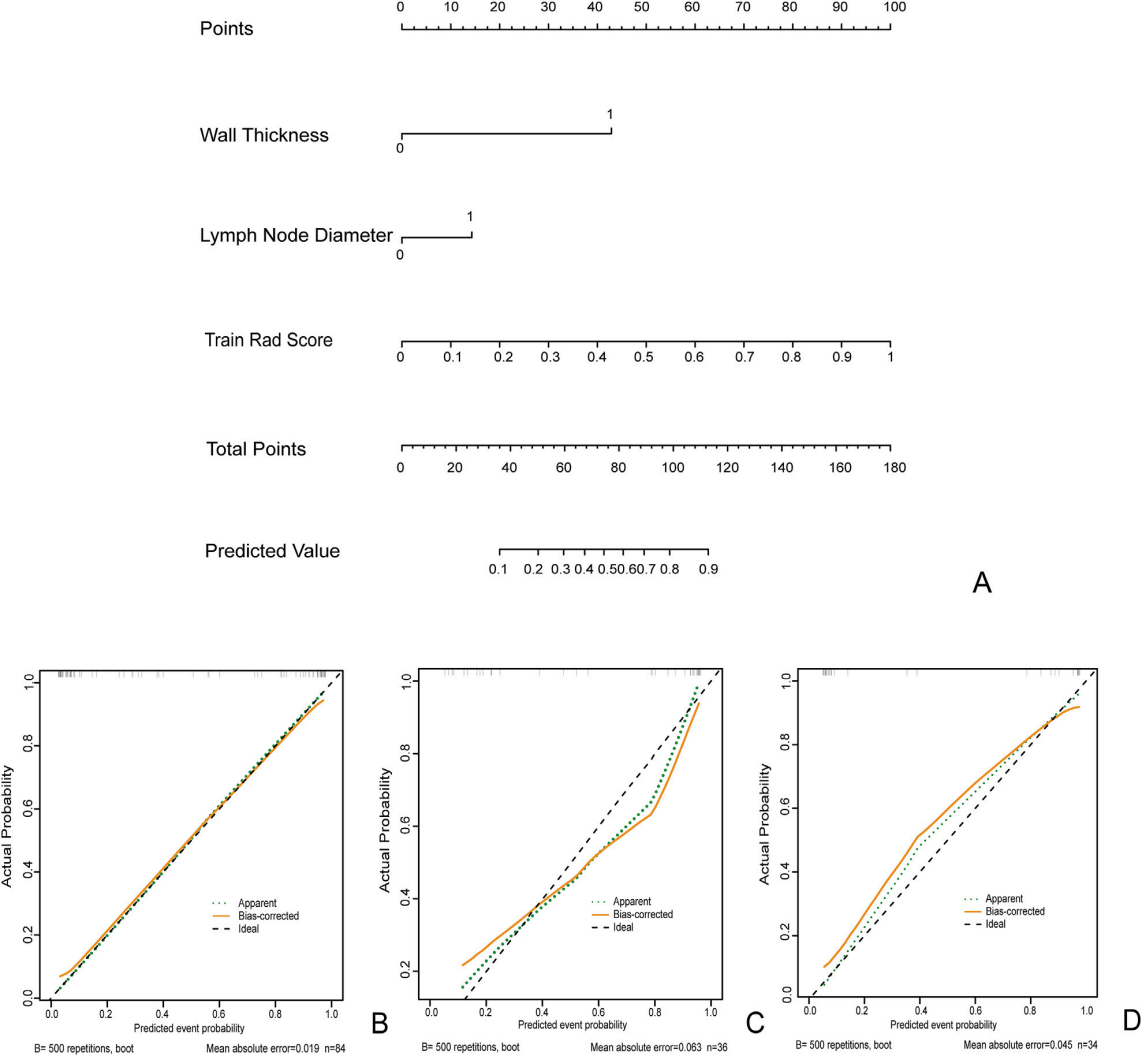
The clinical radiomics nomogram **(A)** for Predicting MH: Intestinal Wall Thickness (0: Intestinal Wall Thickness <10 mm 1: Intestinal Wall Thickness ≥10 mm). Lymph node diameter (0: diameter<10 mm 1: diameter ≥10 mm). Calibration curves for training queue **(B)**, internal validation cohort 1 **(C)**, and internal validation cohort 2 **(D)**.

Use the Nomogram to represent the comprehensive prediction model to improve clinical usability and practicality (Figure 6A).

**FIGURE 7:**
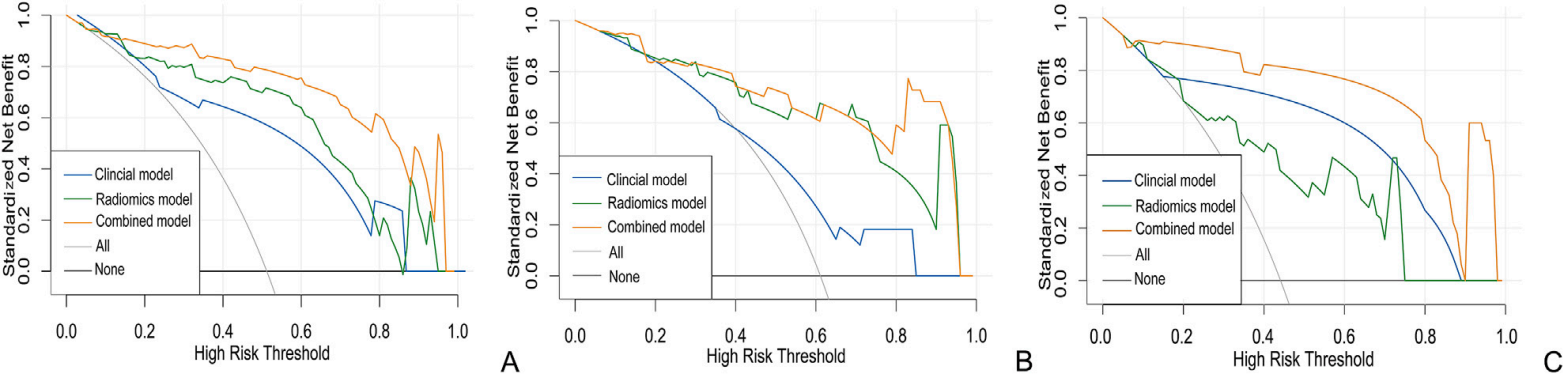
Clinical decision curve of the three models with the training **(A)** internal validation cohort 1 **(B)** and internal validation cohort 2 **(C)**.

**FIGURE 8:**
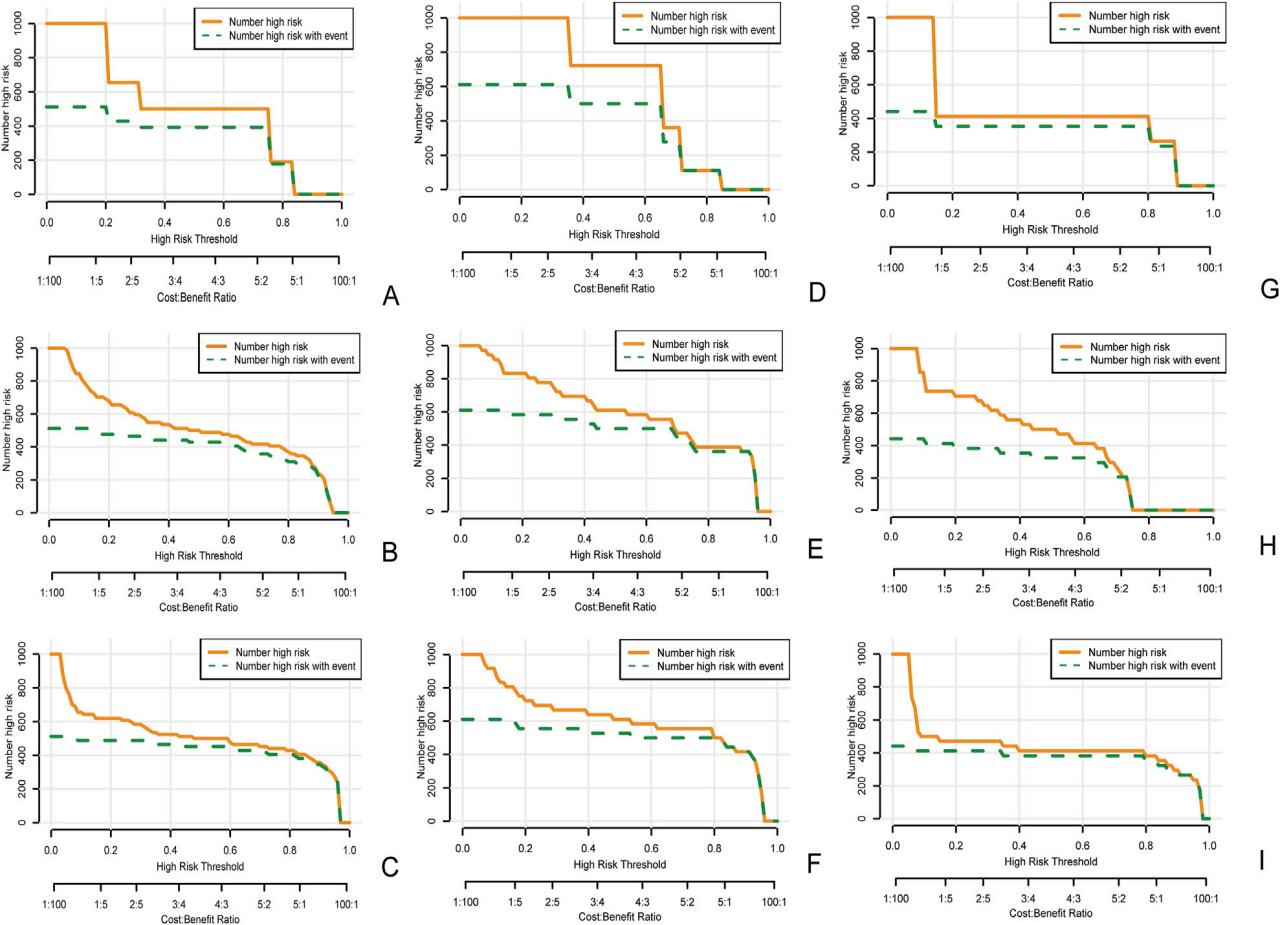
Clinical impact curve of the clinical model **(A)**, the radiomics model **(B)**, and the clinical radiomics nomogram **(C)** in the training cohort, as well as the clinical impact curves of internal validation cohort 1 **(D–F)** and internal validation cohort 2 **(G–I)**. The orange curve represents the number of people classified by the model as mucosal healing at each threshold probability. The green curve represents the true number of people with mucosal healing at each threshold probability. The closer the two curves are, the more accurate the prediction model is.

In the clinical radiomics nomogram, the critical value for distinguishing MH from Non-MH is 0.553 (Figure 5B). In the training cohort, the AUC value, sensitivity, specificity, and accuracy for predicting early MH under endoscopy were 0.948 (95% CI: 0.902–0.995), 0.884, 0.927, and 0.893, respectively. The AUC values, sensitivity, specificity, and accuracy in the internal validation cohort 1 were 0.925 (95% CI: 0.845–1.0), 0.773, 1.0, and 0.833, respectively. In the internal validation cohort 2, the AUC value, sensitivity, specificity, and accuracy were 0.940 (95%CI: 0.802–0.993), 0.933, 0.895, and 0.912, respectively (Table 4; Figure 3). ROC curve analysis (Figure 3) demonstrated that the clinical radiomics nomogram achieved a higher AUC than both the clinical prediction model (p < 0.05) and the radiomics prediction model (P = 0.051), as confirmed by the DeLong test (Table 4). These results suggest that the clinical radiomics nomogram has superior discriminative ability in identifying patients who achieve early MH. Furthermore, the calibration curve (Figures 6B-D) indicated strong agreement between predicted and observed outcomes, supporting the model’s reliability. In addition, to further evaluate its clinical applicability, we constructed a clinical decision curve analysis (DCA; Figure 7), illustrating that the clinical radiomics nomogram provides substantial net benefit across a range of threshold probabilities, and the clinical impact curve (CIC; Figure 8) shows that the predicted results of the clinical radiomics nomogram are closer to the true values, confirming its clinical utility.

## 4 Discussion

In this study, we evaluated a clinical radiomics nomogram combining clinical risk factors and CTE-based radiomics features to predict early MH in CD patients following biologic therapy. Our results demonstrated that while both the clinical prediction model and radiomics prediction model alone could predict early MH in the training and validation cohorts, the clinical radiomics nomogram further improved predictive performance. Moreover, DCA and CIC analysis confirmed the clinical utility of the combined model.

At present, biological therapy plays a crucial role as the frontline clinical management for CD, offering the potential for sustained clinical remission. Since not all the patients correspond well to this biological therapy, it is getting more and more important to find effective ways to predict treatment results. Various efficacy evaluation criteria exist, including clinical symptom remission and the Crohn’s Disease Activity Index (CDAI) score. Compared to other predictive indicators, MH is more objective and is proved to have good relationship to improved clinical symptoms and reduced hospitalization rates of patients (De Cruz et al., 2013; Kang et al., 2016), so we used MH as the evaluation endpoint for short-term efficacy in this retrospective study. Ultimately, we developed a novel nomogram that effectively predicts MH in CD patients undergoing short-term biological therapy, potentially aiding clinicians in optimizing treatment strategies.

As for prediction of MH after biological therapy for CD, many efforts both in clinical and imaging fields have been made and gained some progress. For example, some biological markers, such as a joint hemoglobin, C-reactive protein, fecal calprotectin (FC), plasma interleukin-9 (IL9) and the C-reactive protein/albumin ratio have certain diagnostic and predictive value for MH (Qin et al., 2016; Feng et al., 2017; Weinstein-Nakar et al., 2018; Dreesen et al., 2020; Zhou et al., 2021; Han et al., 2022; Magro et al., 2023; Ye et al., 2023). Therefore, we also incorporated various clinical and routine imaging factors such as C-reactive protein, erythrocyte sedimentation rate, serum albumin, along with imaging features such as intestinal wall thickness, mural stratification, intestinal narrowing, comb sign, lymph node diameter, and perianal lesions. But among the factors, only wall thickness and lymph node diameter were proved as independent factors for predicting treatment effect.

In previous studies of CD, radiomics has been used to evaluate the activity, fibrosis, and differentiation of intestinal tuberculosis in Crohn’s disease (Li et al., 2021; Zhu et al., 2021; Li et al., 2022; Alyami, 2023). In a study to predict treatment efficacy, Li et al. (Shen et al., 2023) followed up patients for at least 1 year through CDAI evaluation and found that combining radiomics with visceral fat can effectively predict the efficacy of Infliximab treatment. Song et al. (2024) predicted the failure of Crohn’s disease patients after at least 1 year of infliximab treatment by combining skeletal muscle index (SMI) and creeping fat with radiomics. In their study, treatment failure was defined as the inability to achieve sustained clinical remission, disease recurrence, or progression within 1 year of drug treatment compared to baseline (CDAI increased to baseline levels or higher, active intestinal inflammation shown on endoscopy or imaging). A point which made our study different from the previous studies is that we used endoscopic MH 3–6 months after treatment as the endpoint, tried to predict the short-term therapy effect to shorten the time window of therapy decision. Though the result showed that our prediction model worked well for short-term treatment effect, further long-term effect for the same group of patients is still needed to prove its stability and consistency.

In another study on predicting MH, Zhu et al. (2022) proposed that a clinical radiomics nomogram, combining radiomics and disease duration, could predict MH after 26 weeks of infliximab treatment. In our research, we added additional imaging features such as intestinal wall thickness, mural stratification, intestinal narrowing, comb sign, lymph node diameter, and perianal lesions beyond the bowel lesion to predict MH. Through univariate and multivariate analyses, we identified intestinal wall thickness and lymph nodes as significant factors in predicting MH. Specifically, when the intestinal wall thickness exceeded 10 mm and lymph node diameter was greater than 10 mm, it suggested that achieving early MH after drug treatment would be challenging for patients. In contrast, mural stratification, intestinal narrowing, comb sign and perianal lesions are not good predictors. However, constructing a clinical model based solely on these two risk factors yielded suboptimal results in predicting the MH state. This aligns with findings from Zhu et al. (2022), emphasizing the limitations of relying solely on clinical features for MH prediction. It is worth noting that the combined Rad-score significantly improved prediction accuracy. Therefore, we constructed a clinical radiomics nomogram by integrating clinical features and radiomics baseline features based on CTE to predict whether CD patients receiving biologic therapy can achieve early MH. Our results indicate that the clinical radiomics nomogram combining clinical and radiomics features can significantly improve its predictive efficiency. The AUC values for the training, internal validation cohort 1, and internal validation cohort 2 are 0.948, 0.925, and 0.940, respectively. The DCA demonstrated that the net benefit of using the clinical radiomics nomogram to evaluate MH far surpasses that of relying solely on clinical or radiomics features. Concurrently, the CIC shows that the predicted results of the clinical radiomics nomogram are closer to the true values. These findings collectively underscore both the clinical feasibility and significant practical utility of the clinical radiomics nomogram in predicting MH outcomes.

There are certain limitations in our study. Firstly, being a retrospective study, it inherently carries potential selection bias. Moreover, the data from the single-center electronic medical record system, combined with issues such as non-standard data entry and low patient compliance, has led to incomplete recording of key demographic variables (smoking status, body mass index (BMI), and disease duration), which may introduce some biases into the study results. Secondly, the manual segmentation of the ROIs for the intestinal wall introduces some variability, despite the conducted consistency testing. This variability might arise from difference in the original morphology of the intestinal wall from inefficient bowel preparation. We chose to repeat multiple times to avoid such errors as much as possible. Secondly, as we have already discussed, we only focused on short-term treatment efficacy in this study, which needs further following-up. Additionally, while our analysis included patients treated with the two main biological agents (infliximab and ustekinumab) and found no significant intergroup differences, potential variations across treatment-strategy centers warrant further investigation. Future studies should aim to validate these findings with larger sample sizes across multiple centers. Finally, the relatively small sample size and lack of multicenter data may compromise the model’s accuracy. To address these limitations, we plan to expand the sample size, conduct multicenter studies, and extend patient follow-up duration in future research. Furthermore, we intend to incorporate advanced techniques such as semi-automatic image segmentation and deep learning to enhance both the predictive depth and accuracy of our models.

## 5 Conclusion

Our study integrated clinical and imaging features with radiomics methods to establish and validate a clinical-radiomics nomogram. The results suggest that radiomics holds significant potential in predicting early MH, providing valuable insights to clinicians for optimizing clinical decision-making in the treatment of Crohn’s disease patients.

## Data Availability

The raw data supporting the conclusions of this article will be made available by the authors, without undue reservation.
